# Resident cells of the myocardium: more than spectators in cardiac injury, repair and regeneration

**DOI:** 10.1016/j.cophys.2017.08.001

**Published:** 2018-02

**Authors:** GA Gray, IS Toor, RFP Castellan, M Crisan, M Meloni

**Affiliations:** 1 BHF/University Centre for Cardiovascular Science, Edinburgh, Scotland, UK; 2 Scottish Centre for Regenerative Medicine, Edinburgh Medical School, The University of Edinburgh, Edinburgh, Scotland, UK

## Abstract

Multiple resident cell types contribute to maintaining the structure and physiological function of the heart over the life course. Cardiomyocyte proliferation supports scar free regeneration in the neonatal heart following injury, but a lower rate of proliferation in the adult necessitates replacement by a collagen scar to maintain ventricular integrity. In this short review we discuss recent studies that have identified novel roles for non-myocyte resident cells and the extracellular matrix in supporting repair, as well as cardiomyocyte and vascular regeneration, following myocardial infarction.

## Highlights

• Cell cycle re-entry accounts for most new cardiomyocytes and endothelial cells in the adult heart.
• The extracellular matrix is a key source of molecules that regulate heart cell behaviour.
• Fibroblasts change their phenotype to resolve inflammation after phagocytosing apoptotic cells.

**Current Opinion in Physiology** 2018, **01**:46–51

This review comes from a themed issue on **Cardiac Physiology**

Edited by **Merry Lindsey** and **David Eisner**

For a complete overview see the Issue and the Editorial

Available online 10th October 2017

**https://doi.org/10.1016/j.cophys.2017.08.001**

2468-8673/© 2017 The Authors. Published by Elsevier Ltd. This is an open access article under the CC BY license (http://creativecommons.org/licenses/by/4.0/).

Given the role of the heart as a muscular pump, cardiac muscle cells, or cardiomyocytes, are clearly key among resident cells. However, the majority of myocardial cells are non-myocytes, including endothelial cells in the coronary vasculature, lymphatics and endocardium, fibroblasts, pericytes, neurons, stem cells and immune cells [1^•^], that each have homeostatic functions to maintain the structure and function of the heart. Cardiac injury following myocardial infarction (MI), is accompanied by necrosis, as well as programmed cell death by apoptosis and necroptosis [2], that reduces cardiac contractile capacity. Adult cardiomyocytes have very limited potential for proliferation, and while this can increase to some extent following injury, the rate is too slow (approx. 0.5–2% per year) to replace the large number of cardiomyocytes lost after MI [3^••^]. Therefore, following cardiomyocyte loss the remaining tissue resident cells variably proliferate, alter their phenotype, transdifferentiate, secrete enzymes, chemical mediators and other intracellular material, for example exosomes [4], to reorganize the matrix and recruit cells necessary for repair. This ensures the formation of collagen scar to maintain the integrity of the heart and its pump function. In this short review we shall focus on recent discoveries with regard to cardiac resident cells, their actions and interactions following injury.

## Cardiomyocytes

Cardiomyocytes account for 20–35% of cells in mouse [5] and human hearts [6], and despite rapid intervention to restore blood supply following MI, significant numbers are lost due to ischemia and to reperfusion injury. Far from being an innocuous event, cardiomyocyte death itself provides the first stimulus for repair by releasing damage associated molecular patterns (DAMPS, or alarmins). These signals activate pattern recognition receptors on neighboring cells, including fibroblasts [6, 7], to initiate recruitment of inflammatory cells. The engulfment of apoptotic cardiomyocytes by macrophages, during efferocytosis [8], and also by resident fibroblast derived myofibroblasts [9^••^], additionally regulates infarct repair by enhancing acquisition of a phenotype that promotes inflammation of resolution.

Various progenitor cell populations can be identified in the heart, although they are relatively rare, and the extent to which they contribute to new cardiomyocyte generation seems to be low [3^••^]. Although still an area of some controversy, the current consensus is that any limited generation of new cardiomyocytes that does occur is predominantly by cell cycle re-entry of existing adult cardiomyocytes [3••, 10]. The challenge is now to understand why the proliferation rate in the adult fails to achieve that which supports full regeneration of the neonatal heart following MI. Interestingly, the extracellular matrix is emerging as an endogenous regulator of cardiac regeneration. In a recent study Basset *et al*. [11^•^] provided evidence for promotion of cardiomyocyte proliferation by extracellular matrix derived agrin, through Yap and ERK mediated signaling, enabling cardiac regeneration in neonatal mice following MI. One of the potential roadblocks to cardiac regeneration is the relatively high pressure within the adult mammalian heart [10]. In this regard it is intriguing that mechanical unloading in humans, following implantation of a left-ventricular assist device, resulted in enhancement of cardiomyocyte proliferation [12]. It is feasible to speculate that the associated changes in mechanical strain might link extracellular matrix signaling to this outcome. Hypoxia also enhances adult cardiomyocyte proliferation, through alteration of redox signaling and mitochondrial mass, and this mechanism was accessed *in vivo* in the adult mouse by exposure to hypoxic environment, resulting in improved outcomes post-MI [13].

## Endothelial cells

Endothelial cells (ECs) make up the largest proportion (60%) of non-myocytes in the adult heart, at least in the mouse [1^•^]. They have a number of essential roles in heart development, in vascular homeostasis, in promoting cardiomyocyte organization and survival, as well as in healing and regeneration post-ischemic injury. Following MI, neovascularization increases the density of peri-infarct vessels thus enhancing perfusion and limiting further loss of cardiomyocytes around the infarct zone. Genetic-lineage tracing has revealed that angiogenesis post-MI occurs preferentially from pre-existing adult ECs, rather than through transdifferentiation from other cell lineages [14]. These new data also suggest that recruited bone marrow derived endothelial progenitor cells might be less important for post-MI angiogenesis than previously proposed. Effective neovascularization following MI requires maturation of nascent vessels through acquisition of a mural coat. Chen *et al.* have now shown that endocardial ECs undergo endothelial to mesenchymal transition (EndMT) to give rise to PDGFRβ+ mural cells (pericytes and vascular smooth muscle cells) during embryonic development [15^•^]. As developmental programmes are frequently re-initiated during remodeling in response to MI or pressure overload, it will be of interest in future to investigate whether neovascularization invokes this pathway, to complement recruitment of resident pericytes during vessel maturation [16]. Investigation of mechanisms for endogenous promotion of angiogenesis continues to identify new pathways that might be exploited to therapeutically enhance angiogenesis in the post-MI setting by acting directly on ECs, for example, CXCR7 [17], micro RNAs [18] and long noncoding RNAs [19], or indirectly via actions in other resident cells, for example, locally regenerated glucocorticoids [20]. The endothelium of lymphatic vessels serves as a barrier to control fluid balance and immune cell trafficking in maintenance of tissue homeostasis. Lymphangiogenesis, the formation of new lymphatic vessels from pre-existing vessels, is also increased post-MI [21]. While in other settings this can have detrimental effects, enhancement in the heart following administration of vascular endothelial growth factor (VEGF)-C was reported to improve structural and functional remodeling post-MI [21]. Promotion of lymphatic vessel maturation by apelin may offer further benefit [22^•^].

Vascular ECs are also a key site for regulation of inflammatory cell recruitment following MI. Senescent ECs have impaired capacity for inflammatory regulation [23], and this may contribute to altered responses to myocardial injury in aging. In addition to generation of mural cells, EndMT, under the influence of TGFβ and loss of signals maintaining the EC phenotype, allows EC to contribute to the fibroblast population in the heart [24]. However, the importance of these cells, relative to resident fibroblasts, in contributing to scar formation remains the subject of debate [25].

## Fibroblasts

Resident fibroblasts are among the most represented cell populations of the heart, although a recent elegant study has shown that the proportion may be <20% in the mouse heart, significantly less than previously suggested [1^•^]. Nevertheless they have a key homeostatic role in synthesis of the cardiac extracellular matrix, and undergo phenotype conversion to proliferative myofibroblasts following MI to augment matrix production, ensuring scar formation [7]. The recent availability of new mouse strains that allow tracking of fibroblast and myofibroblast behavior following myocardial injury has helped to reveal a surprising diversity in their roles [26^•^]. This includes the novel observation that myofibroblasts phagocytose apoptotic cells in the heart following MI [9^••^]. Knockout of the milk fat globule-epidermal growth factor 8, that is secreted by myofibroblasts to enable phagocytosis, resulted in impaired clearance of apoptotic cells, and increased mortality [9^••^]. (Myo) fibroblasts are an important source of inflammatory mediators in the heart, including those responsible for neutrophil recruitment [7, 27]. The fibroblast phenotype is determined by inputs from other cells in the microenvironment within the heart, for example DAMPS, and interleukin (IL)-1 that promote inflammation, and apoptotic cells that are anti-inflammatory. Crosstalk with ECs and with macrophages ensures promotion of angiogenesis and matrix synthesis. There is much still to learn about cardiac (myo) fibroblasts [28], and the ability to target genetic modification to fibroblasts or specifically to myofibroblasts [29], will undoubtedly lead to greater understanding of their roles and interactions during wound repair. Transdifferentation of fibroblasts to ECs can occur during mesenchymal to EC transition (MEndT) *in vitro*, but whether this contribute significantly to EC generation *in vivo* is less clear [14]. *In situ* reprogramming of cardiac fibroblasts to cardiomyocytes by administration of transcription factors or microRNAs has generated excitement in the regenerative medicine field, and the search for small molecule alternatives to allow pharmacological intervention holds much promise for translation of this approach [30].

## Pericytes

**Figure 1:**
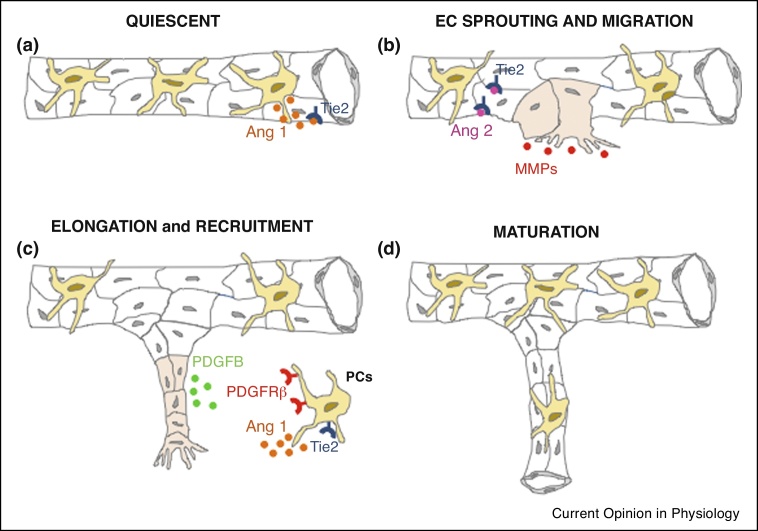
New blood vessel formation and maturation requires communication between endothelial cells (ECs) and pericytes (PCs) through paracrine factors. **(a)** A quiescent capillary: cell–cell contact between ECs (in white) and PCs (in yellow) maintains capillaries in a quiescent state, in part through the actions of pericyte derived angiopoetin 1 (Ang 1) on EC Tie 2 receptors. **(b)** Cardiac injury (MI) triggers neovascularisation (from pre-existing adult ECs — in pink) and ECs release Ang 2 that prevents access of Ang 1 to EC Tie 2 receptors and allows EC sprouting and pericyte detachment. Sprouting ECs also release MMPs that promote pericyte detachment and EC migration. **(c)** PDGFB is released by ECs during the elongation process. Pericytes expressing the PDGFRβ are recruited to stabilize and mature the new vessels **(d)**. Maturation is also promoted by the binding of Ang 1 to pericyte Tie 2 receptors.

Pericytes are smooth muscle like cells of mesenchymal origin that surround capillary ECs of the heart, and also have multipotent progenitor potential [31]. Cell–cell contact between pericytes and EC maintains them in a quiescent state, and initiation of angiogenesis requires the detachment of pericytes to enable EC migration (Figure 1). Vice versa, pericyte recruitment stabilizes and matures nascent vessels. In a new study, Teichert *et al*. [16] have revealed an essential role for angiopoietin/Tie 2 signaling in regulating these interactions via the Tie 2 receptor expressed on pericytes, in addition to ECs. In other tissues, pericytes are important regulators of immune cell recruitment [32, 33•, 34, 35], and they are also likely to have this role in the heart following MI. Pericytes are progenitors of multiple cell types *in vitro*, and when administered to the mouse heart *in vivo* following MI can contribute to development of new cardiomyocytes, albeit in limited manner [31]. Pericytes can also assume a collagen synthesizing phenotype and contribute to tissue fibrosis [36]. The extent to which pericytes behave as mesenchymal cells *in vivo* is the subject of some controversy [37], and further lineage tracking studies are required to investigate the roles of these cells during repair and regeneration in the heart.

## Immune cells

Immune cells, including monocytes and neutrophils, are rapidly recruited in large numbers to the heart following injury [38], but there is also a significant resident representation before injury, including macrophages and small populations of B and T cells, in the mouse heart [1^•^].

Mast cells, long established as resident cardiac immune cells, function as key effectors of the innate immune response and their strategic perivascular location allows preformed stores of inflammatory mediators to be released into the blood when they rapidly degranulate following MI [38]. Recent studies have shown that mast cell derived renin activates the local renin-angiotensin system [39], and mast cell derived chymase can degrade insulin-like growth factor-1 [40], increasing ischemic cardiac injury and detrimental remodeling following MI.

**Figure 2:**
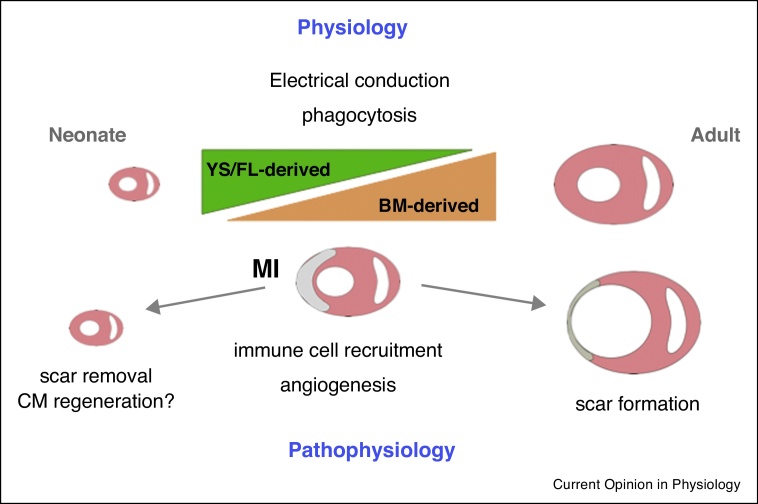
Roles of resident macrophages in physiology and in pathophysiology following MI. Yolk-sac and fetal liver (YS/FL) derived macrophages that predominate in the neonatal mouse heart are gradually replaced over the lifetime by bone-marrow (BM) derived macrophages that may provoke a greater inflammatory response to injury. Macrophages have a phagocytic role in the healthy mouse heart and in the atrio-ventricular conducting system are required for electrical signaling. Following MI, macrophages are required for angiogenesis in neonatal and adult mice, and may have a role in regulating cardiomyocyte proliferation under hypoxic conditions, at least in neonates. Macrophages are important for scar removal in neonatal mice to ensure scar free regeneration, but influence fibroblast activation in the adult to ensure formation of a replacement scar in the absence of efficient regeneration.

Although a resident macrophage population (Figure 2) has only relatively recently been described in the heart [41, 42], it is the subject of intense current scrutiny with regard to roles in physiology and pathophysiology [43]. Originating from the fetal yolk sac and liver, with an increasing contribution from bone marrow derived cells in the adult [44] (Figure 1), the resident cardiac macrophage population is relatively sparse in healthy hearts, and phagocytically active, consistent with a janitorial homeostatic role [42, 45]. Identification of increased macrophage density in the atrio-ventricular conducting system has recently led to discovery of an unexpected role in facilitation of electrical conduction in the heart [46^••^]. Following MI, resident macrophages [47], alongside fibroblasts [27], release chemoattractant molecules that guide neutrophil recruitment to clear necrotic cardiomyocytes. Given these key roles for resident macrophages it will be interesting to know whether phenotypic changes in response to age, obesity or systemic inflammation influence the likelihood of arrhythmia or the early inflammatory response following MI. In the neonatal mouse heart, macrophages are required for cardiac regeneration, where their role is to support vascularization and scar resolution [48], although it has been suggested that they also promote cardiomyocyte proliferation under conditions of hypoxia [49]. Resident cardiac macrophages do not proliferate *in situ* in response to a Th2 immune stimulus [45], unlike resident populations in some other tissues, and are rapidly outnumbered by macrophages derived from recruited monocytes soon after MI [41]. An important area for future investigation will be the status of the resident macrophage population after inflammation resolution and how this influences longer term cardiac remodeling and the response to subsequent cardiovascular insult, be it ischemia or pressure overload.

## Future directions

As our understanding of the molecular mechanisms involved in myocardial injury, repair and regeneration increases what emerges is a picture of integrated signaling among the multiple resident cell types of the myocardium, and between these cells and those recruited to the heart. The extracellular matrix is coming to the fore [50] with its ability to communicate changes in biomechanical strain and to secrete molecules that influence the cells that it surrounds. The microenvironment in the infarct, peri-infarct and remote myocardium varies during injury and repair and determines the phenotype and activation status of cells including fibroblasts, endothelial cells and macrophages, ensuring progression from removal of dead cells to their replacement by scar in the adult, or by new myocardial tissue in the neonate. Aging and co-morbidities such as obesity and diabetes will undoubtedly influence these cellular interactions. As we move closer to effective enhancement of cardiomyocyte proliferation in the adult heart, the challenge will be to bring these elements together so that we can better understand how to promote myocardial regeneration and scar removal, while maintaining the integrity and pump function of the heart. Advances in molecular imaging [51], single cell sequencing and *in silico* modeling of biological processes [52] may best provide the means to achieve this end.

## Funding

The authors were supported by research grants from Wellcome Trust (WT104799/Z/14/Z) and the British Heart Foundation (BHF FS/12/65/30002 and CH/09/002/26360) and by British Heart Foundation Centre of Research Excellence funding.

## Conflict of interest

Nothing declared.

## References and recommended reading

Papers of particular interest, published within the period of review, have been highlighted as:

• of special interest
•• of outstanding interest
